# Cortical monitoring of cardiac activity during rapid eye movement sleep: the heartbeat evoked potential in phasic and tonic rapid-eye-movement microstates

**DOI:** 10.1093/sleep/zsab100

**Published:** 2021-04-17

**Authors:** Péter Simor, Tamás Bogdány, Róbert Bódizs, Pandelis Perakakis

**Affiliations:** 1 Institute of Psychology, ELTE, Eötvös Loránd University, Budapest, Hungary; 2 Institute of Behavioural Sciences, Semmelweis University, Budapest, Hungary; 3 UR2NF, Neuropsychology and Functional Neuroimaging Research Unit at CRCN – Center for Research in Cognition and Neurosciences and UNI – ULB Neurosciences Institute, Université Libre de Bruxelles (ULB), Brussels, Belgium; 4 Doctoral School of Psychology, ELTE Eötvös Loránd University, Budapest, Hungary; 5 National Institute of Clinical Neurosciences, Budapest, Hungary; 6 Department of Social, Organisational, and Differential Psychology, Complutense University of Madrid, Madrid, Spain; 7 Brain, Mind, & Behavior Research Center, University of Granada, Granada, Spain

**Keywords:** heartbeat evoked potential, sleep, REM, interoception, microstates

## Abstract

Sleep is a fundamental physiological state that facilitates neural recovery during periods of attenuated sensory processing. On the other hand, mammalian sleep is also characterized by the interplay between periods of increased sleep depth and environmental alertness. Whereas the heterogeneity of microstates during non-rapid-eye-movement (NREM) sleep was extensively studied in the last decades, transient microstates during rapid-eye-movement (REM) sleep received less attention. REM sleep features two distinct microstates: phasic and tonic. Previous studies indicate that sensory processing is largely diminished during phasic REM periods, whereas environmental alertness is partially reinstated when the brain switches into tonic REM sleep. Here, we investigated interoceptive processing as quantified by the heartbeat evoked potential (HEP) during REM microstates. We contrasted the HEPs of phasic and tonic REM periods using two separate databases that included the nighttime polysomnographic recordings of healthy young individuals (*N* = 20 and *N* = 19). We find a differential HEP modulation of a late HEP component (after 500 ms post-R-peak) between tonic and phasic REM. Moreover, the late tonic HEP component resembled the HEP found in resting wakefulness. Our results indicate that interoception with respect to cardiac signals is not uniform across REM microstates, and suggest that interoceptive processing is partially reinstated during tonic REM periods. The analyses of the HEP during REM sleep may shed new light on the organization and putative function of REM microstates.

**Statement of Significance**

Rapid-eye-movement (REM) sleep is a heterogeneous sleep state characterized by transient periods with (phasic REM) and without (tonic REM) eye movements. Phasic and tonic REM periods differ in spontaneous cortical activity, awakening thresholds, and the processing of external inputs (exteroception). To date, no previous studies have explicitly investigated the processing of internal (or endogenous) signals during phasic and tonic REM microstates. In this study, we examined the heartbeat evoked potential, a cortical response to heartbeats reflecting interoceptive processing. Our findings show that interoceptive processing is differently modulated by phasic and tonic REM microstates, and indicate that interoceptive processing is partially reinstated in tonic REM periods.

## Introduction

Sleep is a remarkably heterogeneous state expressing two antagonistic needs: to undergo neural recovery under environmental disconnection, and to maintain sensory processing in order to monitor external inputs to some extent [1–3]. Such variability gives rise to intermittent transitions between stable sleep states and periods of increased susceptibility to external inputs [1, 3–5]. While significant research efforts have focused on the continuous fluctuations between stable sleep states and brief arousals in NREM sleep [6–8], the microarchitecture of rapid-eye-movement (REM) sleep has received less attention.

REM sleep is a fundamental state that occupies around 20% of nighttime sleep in healthy human adults and is associated with a variety of functions from basic physiological mechanisms, to complex cognitive phenomena [9]. Although REM sleep is usually treated as a homogeneous sleep state, it is composed of two remarkably different microstates: phasic and tonic REM sleep. Phasic REM periods feature bursts of eye movements related to ponto-geniculo-occipital (PGO) waves [10], muscle atonia interspersed with brief muscle twitches, and irregularities in respiratory and cardiac activity [11], whereas tonic periods are more quiescent epochs without eye movements.

Recent studies suggest, that the alternation of phasic and tonic periods might reflect transient fluctuations between sensory detachment and more externally focused (off-line vs. on-line) states, respectively. For instance, neural activity during phasic periods seems to be detached from the surroundings, indicating internally driven sensorimotor processing [12, 13], and the activity of a functionally isolated, thalamocortical network [14, 15]. In contrast, the brain during tonic REM appears to be more responsive to external stimuli as evidenced by awakening and arousal thresholds [16, 17], as well as by auditory evoked blood-oxygen-level-dependent (BOLD) responses [15] and electroencephalography (EEG) [18, 19]. For instance, event-related potentials (ERPs) elicited by deviant stimuli (i.e. an infrequent tone embedded in frequent ones) consistently showed preserved cortical responses during tonic REM resembling evoked potentials in wakefulness, whereas such responses were not detected in phasic REM periods [19–21]. These findings may suggest that phasic REM is a microstate characterized by sensory detachment, while tonic REM protects the organism from potential external dangers by reinstating, to some extent, environmental alertness [22].

In sum, the available empirical evidence seems to indicate that the neural processing of external environmental stimuli is relatively enhanced in tonic REM sleep; however, no previous studies have explicitly investigated the processing of bodily (e.g. visceral) signals during phasic and tonic REM microstates. Indeed, a rich source of information for the sleeping brain is arising from the body through a stream of afferent signals whose neural processing is known under the term interoception [23, 24]. For instance, the processing of nociceptive stimuli is somewhat attenuated after falling asleep, but do persist to some extent in all sleep stages, including REM sleep [25]. Moreover, cortical responses to nociceptive inputs predicted arousals [26] in line with the assumption that interoceptive signals may profoundly influence the regulation of sleep and arousal [1]. Therefore, we may assume that beyond exteroception, interoceptive processing is also distinguished across REM microstates.

We examined interoception, more specifically the cortical monitoring of cardiac activity in phasic and tonic REM states by focusing on the heartbeat evoked potential (HEP) [27–29]. The HEP is an EEG potential that reflects the cortical processing of afferent cardiac signals, and is quantified by averaging the scalp EEG potentials time-locked to the R-peak of the individual heartbeats [28]. The amplitude of the HEP is positively correlated with the ability to perceive one’s own heartbeats [27, 30, 31]; however, it is also observed during rest, when individuals are not instructed to focus on their heartbeats [32, 33], or in sleep [34, 35], in particular, during the REM stage [36, 37]. On the other hand, the amplitude of the HEP is slightly modified in conditions of increased arousal and vigilance in a state-and trait-like manner.

More specifically, different vigilance states during sleep were associated with differences in HEP amplitudes [36] that showed gradual changes from wakefulness to deeper sleep stages (Stage 2 and slow-wave sleep). On the other hand, HEPs in more aroused sleep stages such as Stage 1 and REM sleep resembled HEPs in wakefulness [35, 36]. Likewise, inducing arousal during wakefulness also led to modulations in HEP amplitudes, that according to the authors indicated increased interoceptive processing during periods of increased arousal [38]. Moreover, insomnia patients (featuring signs of hyperarousal) also exhibited different HEPs before falling asleep, compared to a control group of good sleepers [39].

Since tonic compared to phasic REM periods appears to exhibit increased environmental alertness and maintained neural responses to sensory inputs, we anticipated that HEP amplitudes would be modulated differently by phasic and tonic REM microstates. Moreover, several studies indicate that neural activity in tonic REM appears as an intermediate state between phasic REM and resting wakefulness [13, 15, 40]. These findings indicate that although sensory processing is also reduced in tonic REM (i.e. compared to wakefulness) it is nevertheless, reinstated to some extent allowing the appearance of neural responses in reaction to external inputs [15, 19, 20]. Therefore, we hypothesized that HEPs will not only be different in tonic compared to phasic REM, but tonic REM will also resemble the pattern found in resting wakefulness. We report the results from the analysis of two distinct databases, in order to uncover possible differences in the neural processing of individual heartbeats between phasic and tonic REM sleep. In an additional step, we aimed to verify how differences in HEPs during REM microstates relate to the HEP waveform of resting wakefulness. To the best of our knowledge, this is the first study aiming to investigate whether the enhanced environmental alertness observed during tonic REM sleep [15, 21, 41] is also accompanied by a distinct pattern of internal signal processing compared to phasic REM.

## Methods

### Participants and procedure

We performed a sequential analysis of the whole-night polysomnographic recordings of two different databases comprising data from 40 healthy individuals (*N* = 20 in Study 1, and *N* = 20 in Study 2) that served as control participants in two previous studies of our group [42, 43].

In Study 1, individuals (*N* = 20, 10 males, mean age = 21.72 ± 1.36 years) were recruited from three different Hungarian universities for partial credit points. Participants did not report prior or current neurological, psychiatric or sleep disorders or any kind of chronic disease, intake of medicine (except contraceptives), and showed no signs of anxiety or depression based on standard psychometric measurements [44, 45]. Participants were invited to spend two consecutive nights in the sleep laboratory of the Semmelweis University equipped with standard polysomnography [46]. The first night served as an adaptation night, and we restricted our analyses to the recordings of the second night. Participants were not allowed to drink alcohol on the day and the previous day of the examination. They were also asked to avoid napping and consuming caffeine on the afternoon of the measurements. Lights-off were scheduled between 23:00 pm and 01:00 am depending on participants’ habitual bedtimes. We woke up participants after 8 h of undisturbed sleep unless participants woke up earlier spontaneously.

The participants of Study 2 (*N*= 20, six males, mean age = 21.55 ± 1.57 years) were recruited from a pool of students of the Budapest University of Technology and Economics and the Eötvös Loránd University, and through advertisements in social media. Exclusion and inclusion criteria as well as the recording protocol (two consecutive nights assessed with polysomnography) were the same as in Study 1, but the experiment took place at the sleep laboratory of the Budapest University of Technology and Economics. In Study 2, participants were allowed only 7 h of undisturbed sleep, and awakenings were scheduled between 7:00 and 8:00 am unless participants woke up earlier spontaneously. On the second night, participants were shown a set of negative and neutral IAPS (International Affective Picture System; [47] pictures before they went to bed. Participants were asked to provide subjective evaluations (valence and arousal), while physiological data (skin conductance response and heart rate) were collected during the presentation of the images. The procedure and the results of these measurements will be reported elsewhere (Blaskovich et al. in preparation). Here, we only focus on the nocturnal polysomnographic recordings of the second nights. Both studies were approved by the Ethical Review Boards of the corresponding universities and written informed consents were obtained.

### EEG recordings and preprocessing

In Study 1, participants were fitted with gold-coated (Ag/AgCl) scalp EEG electrodes fixed with EC2 Grass Electrode Cream (Grass Technologies, Warwick, Rhode Island, USA). Nineteen scalp derivations (Fp1, Fp2, F7, F3, Fz, F4, F8, T3, C3, Cz, C4, T4, T5, P3, Pz, P4, T6, O1, O2 referenced to the mathematically linked mastoids) were placed according to the standard 10–20 system [46]. In addition, bipolar polygraphic signals were recorded as follows: EOG with electrodes placed above and below the left and the right canthi, respectively, submental EMG and bipolar ECG with electrodes placed on the left and the right chest. Impedances were kept below 8 kΩ. Signals were collected, prefiltered (0.33–1500 Hz, 40 dB/decade antialiasing hardware input filter), amplified, and digitized with 4096 Hz/channel sampling rate (synchronous) with 12-bit resolution by using the 32-channel EEG/polysystem (Brain-Quick BQ 132S; Micromed, Mogliano Veneto, Italy). Finally, the digitized and filtered EEG was downsampled at 1024 Hz. In Study 2, we followed the same recording protocol, but we used only seventeen scalp EEG locations (frontopolar channels were not used), and signals were recorded with Micromed SD LTM 32 Bs (Micromed S.p.A., Mogliano Veneto, Italy) and SystemPLUS 1.02.1098 software (Micromed Srl, Roma, Italy). EEG data was prefiltered (0.33–1500 Hz; 40 dB/decade anti-aliasing hardware input filter), amplified, and digitized with 4,096 Hz/channel sampling rate with 16-bit resolution, and downsampled at 512 Hz sampling rate.

### Selection of phasic and tonic segments

Sleep stages were previously scored manually according to standardized criteria of the American Academy of Sleep Medicine (AASM) [48] by an expert trained in sleep research that was blind to the aims of the present study. Phasic and tonic segments from all epochs scored as REM sleep were categorized by visual inspection based on the presence or absence of a burst of eye movements (EMs). In both databases, four-second-long segments were coded as phasic periods when the EOG channel showed at least two consecutive eye movements that last less than 500 ms and exhibited 100 μV (or larger) amplitudes within the specific time window. Visual inspection was aided by an automatic procedure highlighting eye movement deflections above the predetermined threshold. Bursts of eye movements present on the (0.5–30 Hz) band-pass filtered EOG channel were automatically detected, and amplitudes exceeding the threshold (100 μV) were highlighted.

Previous studies used less conservative amplitude criteria to identify EMs [49, 50]; however, EMs in bipolar EOG montages produce higher amplitudes; therefore, we restricted our analyses to relatively larger EMs. The criterion for the duration of EMs was based on earlier studies indicating that EMs during REM does not exceed 2 Hz in terms of frequency [49, 50]. Tonic segments were defined as four second long segments without significant bursts of eye movements (EOG deflection of less than 25 μV) within the time window (see Supplementary Material, S5). To avoid contamination between the two microstates, segments were only selected if they were at least 8 s apart from each other. The selection of segments were carried out by research assistants trained in sleep scoring, and the selected four seconds long periods were visually inspected by a trained sleep researcher in order to exclude segments with inaccurate categorizations, as well as periods containing movement-related and technical artifacts. In Study 1, we randomly selected 100 segments (400 s) from each state for HEP analyses. In Study 2, the amount of phasic and tonic segments was more variable, but was set to a minimum duration of 6 min in each condition. In case of Study 2, in one participant we could only identify a reduced number (<200) of artifact-free trials, therefore, the participant’s data were not included in the HEP analyses.

### Selection of awake segments

We additionally aimed to contrast HEPs in REM phasic and tonic microstates with the HEP waveforms obtained during resting wakefulness. More specifically, our aim was to contrast the HEPs of awake periods focusing on the time range that differentiated HEPs in phasic and tonic REM. Therefore, we included in our analyses the awake periods before sleep onset, as well as wake segments after sleep onset if the pre-sleep period was too short. Awake segments included exclusively wakeful periods spent with closed eyes. Awake segments were selected and preprocessed in the same manner as REM periods, yielding an average number of 268.95 ± 156.41 awake trials in Study 1 (*N* = 20) and 499.12 ± 175.3 awake trials in Study 2 (*N* = 16; i.e. the awake data of three additional participants were excluded due to the low number of trials).

### Independent component analyses and R peak detection

Data analyses were carried out with MATLAB (version 7.10.0.499, R2010a, The MathWorks, Inc., Natick, MA), using the EEGLAB [51], Fieldtrip [52] and HEPLAB [53] toolboxes, as well as custom-made scripts. EEG data were band-pass filtered between 0.5 and 35 Hz (fourth-order, two-pass, Butterworth, infinite inverse response filter). Independent component analysis (ICA) of the concatenated phasic REM, tonic REM and resting wake segments was performed to identify eye movement artifacts using EEGLAB and Fieldtrip routines [51, 52]. Independent components (mostly two, maximum four) representing components linked to rapid eye movements were detected semi-automatically and were identified by inspecting the waveforms during the three vigilance states, as well as their topographical distribution [54] (see the provided example in Supplementary Material, S1). The HEPLAB toolbox was used for the semi-automatic detection of the R peaks of the ECG signal within the previously selected segments, and the creation of the corresponding EEG trials. The EEG and the ECG signals were segmented to epochs of 1000 ms, extending between –200 ms and +800 ms time-locked to the R peaks, yielding to an average number of 309.45 ± 40.46 trials in Study 1, and 782.42 ± 370.25 trials in Study 2. Phasic and tonic trials were averaged for each participant after baseline correction extending between –200 ms and –50 ms in order to avoid the influence of the rising edge of the R wave [55].

### HEP analysis

Statistical analyses contrasting phasic and tonic conditions were restricted to a time window between 350 ms and 650 ms after the R peak. Although this time window of interest is slightly delayed compared to some earlier descriptions of the timing of HEPs [31, 56], we focused on this period because: (1) cardiac field artifacts originating from the R and the T wave are highly unlikely to contaminate such late potentials [28, 57], (2) HEP-like waveforms were identified in late components during REM sleep [37], (3) conditions of increased vigilance [39], arousal [38], and attentional focus [30, 58] also seem to have an effect on the amplitude of late HEP components. Comparisons between phasic and tonic conditions were performed by cluster-based, nonparametric statistics as implemented in the Fieldtrip toolbox [59]. This statistical procedure is widely used for the analyses of EEG data as it does not involve background assumptions of data distribution, and is able to handle efficiently the issue of multiple comparisons [59]. In brief, two-tailed paired sample *t*-tests were performed for all pairs of data points in time and space. Due to the differences in sampling rate between the studies (1024 vs. 512 Hz), the time axis extending between 350 and 650 ms consisted of steps of approximately 1 and 2 ms, in Study 1 and Study 2, respectively. Clusters were defined if adjacent time points or electrode locations (at least two neighboring channels) showed significant differences at a two-tailed ⍺ level below 0.05. These observed clusters were selected to compute the observed cluster statistic defined by the sum of all the *t*-values that formed a given cluster. The same process was repeated 5,000 times by randomly shuffling phasic and tonic conditions (using Monte-Carlo simulations). From these simulations the largest clusters were extracted in order to create a distribution of the maximal clusters produced by chance. Finally, the observed cluster statistics were tested (with an alpha value of 0.05) against the probability distribution of the largest simulated clusters.

Although ICA correction is a powerful technique to attenuate ECG artifacts, it is nonetheless unable to fully eliminate artifacts arising from the R and the T waves, and more importantly, it is in fact problematic in case of HEP analyses, since it may remove genuine HEP components of the signal [28]. Since cardiac field artifacts were reported to reduce to <1% compared to the ECG signal measured at the chest [57], we decided to control for the confounding effects of ECG artifact following the recent guidelines of Park and Blanke [28] instead of removing cardiac artifacts by ICA. Therefore, we included an additional step to control for the confounding influence of cardiac artifacts by contrasting the ECG signals in the two experimental conditions (phasic and tonic REM periods). More specifically, we extracted the individual average amplitude of ECG potentials within the time window where significant HEP differences were found. We compared the averaged ECG amplitudes within the time window of interest across phasic and tonic REM states with paired samples *t*-tests or Wilcoxon signed-rank tests if the assumption of normality was not fulfilled. In addition, we aimed to verify whether the effect of condition on mean HEP amplitudes (of the specific spatial and temporal cluster) remains significant regardless of the difference in phasic and tonic ECG amplitudes (for the same temporal cluster). Therefore, we performed a repeated-measures analyses of covariance (ANCOVA) model regressing averaged HEP amplitudes by condition (phasic vs tonic) as a within-subject factor and the difference between phasic and tonic mean ECG amplitudes as a covariate (continuous variable) in the equation. Moreover, we tested if the contrasts of the mean of HEP amplitudes (phasic HEP – tonic HEP) and the contrasts of the mean ECG amplitudes (phasic ECG – tonic ECG) were correlated within the extracted time window. Finally, the averages of ECG signals (time locked to the R peak) were also contrasted across phasic and tonic conditions through the entire time range (from –200 to +800 ms post-R) by non-parametric permutation statistic applied on the single ECG channel. In brief, we performed paired *t*-tests at each time point of the averaged ECG potentials. These observed *t*-tests for each time point were then contrasted against the distribution of *t*-values (for the respective time points) obtained from the comparison of 5,000 simulated samples in which phasic and tonic conditions were shuffled. In order to detect any sign of potential confounding ECG activity on HEP differences, both uncorrected and False Discovery Rate (FDR) corrected *p*-values [60] were evaluated in this case.

### Surrogate analysis

In order to verify whether the identified differences across REM microstates are specifically locked to heartbeats or merely reflect differences in oscillatory activity between phasic and tonic REM [61], we performed the same analyses on 100 surrogate datasets following previously recommended procedures [37, 62, 63]. In brief, we created for each participant surrogate R-peaks with similar mean rate and inter-beat intervals as the original R-peaks, but occurring at randomized intervals during phasic and tonic REM periods, separately. This way, based on these surrogate R-peaks new phasic and tonic trials were selected from the clean, artifact-free 4 s long data segments. Trials were selected in the same manner as described above (i.e. 1000 ms long trials were selected and averaged between –200 and 800 ms time-locked to the surrogate R-peaks), and statistical comparisons between the three vigilance states were performed by cluster-based permutation statistics and Monte-Carlo simulation of 5,000 samples shuffled across conditions (similarly to the procedure described in the HEP analysis section). We repeated the same procedure 100 times to obtain the sum of *t*-values that formed clusters. The aim of this procedure was to compare the distribution of cluster statistics (sum of *t*-values) we obtained from the statistical comparisons of the surrogate datasets with the cluster statistics of the original HEP analyses. If the original sum of *t*-values were greater than the five largest sums of *t*-values of the distribution obtained from the analyses of surrogate data, the probability that the differences between the conditions were not specifically related (locked) to the heartbeats is considered to be low (5%).

## Results

### Sleep architecture and cardiac activity in phasic and tonic REM

Sleep efficiency and consensual indices of sleep architecture did not deviate from standard norms for similar age groups (Table 1) [64]. Sleep efficiency was high and participants spent a relatively long time in REM sleep in both studies. Sleep architecture did not differ considerably across studies; however, significant differences (*p* < 0.001) emerged with respect to the percentage spent in Stage 2 and slow-wave sleep (SWS) that can be attributed to the difference in the amount of sleep the participants could spend in the laboratory (i.e. Participants in Study 2 were woken up after 7 h of sleep, yielding a higher relative amount of SWS as this sleep stage predominates during the first half of the night). The average heart rate (HR) during REM microstates was in the normal range in Study 1 (46–77) and Study 2 (49–73), and did not significantly differ across phasic and tonic REM in Study 1 (HR phasic: 61.75 ± 7.5, HR tonic 61.8 ± 6.6, *t*(1,18) = −0.06, *p* = 0.94), and Study 2 (HR phasic: 60 ± 5.01, HR tonic 60.25 ± 5.76, *t*(1,19) = −0.44, *p* = 0.66). Heart rate variability (HRV) was quantified by the standard deviation of the beat-to-beat intervals (SDNN), and due to the differences in the number of samples was normalized with the available number of RR intervals. (Since data was chunked into 4-sec long, not necessarily continuous segments, other metrics such as the HF or RMSSD were not feasible in our case.) In Study 1, phasic periods exhibited significantly lowered heart rate variability as measured by the SDNN values (phasic: 52.38.71 ± 14.97, tonic: 62.15 ± 16.72, *t*(1,19) = −4.98, *p* < 0.001). On the other hand, the SDNN was not significantly different across phasic and tonic segments in the database of Study 2 (phasic: 49.71 ± 13.42, tonic: 52.37 ± 16.2, *t*(1,18) = −1.24, *p* = 0.22).

**Table 1.** Sleep architecture in the two studies

	Study 1 (*N* = 20)	Study 2 (*N* = 20)
	Mean (*SE*)	Mean (*SE*)
Sleep efficiency (%)	94.83 (1.12)	93.94 (1.2)
WASO (min)	18.98 (5.87)	17.52 (5.12)
Sleep latency (min)	7.1 (1.53)	11.38 (2.16)
Wake (%)	5.16 (1.12)	6.06 (1.2)
Stage 1 (%)	2.73 (0.37)	2.18 (0.35)
Stage 2 (%)	49.3 (1.13)	46.8 (0.88)
SWS (%)	18.03 (1.04)	23.88 (0.76)
REM (%)	24.78 (1.03)	21.08 (0.78)
REM latency (min)	84.47 (7.71)	94.96 (8)

WASO, wake after sleep onset; SWS, slow wave sleep; REM, rapid eye movement sleep.

### HEP differences in Study 1

**Figure 1.**
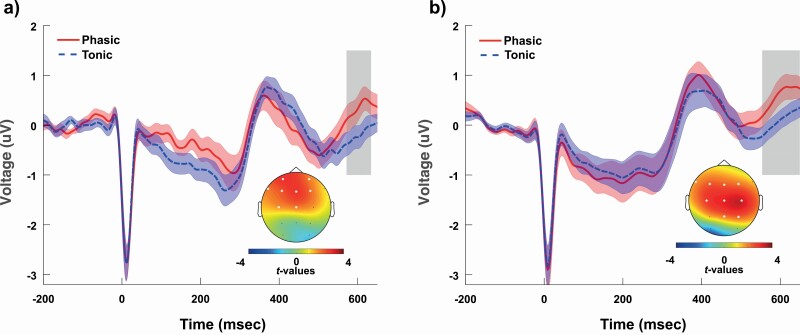
HEP (heartbeat evoked potential) waveform during phasic and tonic REM sleep in Study 1 (A) and Study 2 (B). HEP amplitudes differed at a trend level in Study 1 (cluster level *p* = 0.0442 between 571.5 and 634 ms) and showed significant differences in Study 2 (cluster level *p* = 0.006 between 553.9 and 649.6 ms). Clusters in time and space were extracted and corrected for multiple comparisons by cluster-based permutation statistics (Maris and Oostenveld, 2007). The headplots illustrate the channel locations that formed the clusters within the time range shaded by gray rectangles. The lineplots indicate the HEP waveforms and standard errors averaged across the channels that belonged to the clusters.

**Figure 2.**
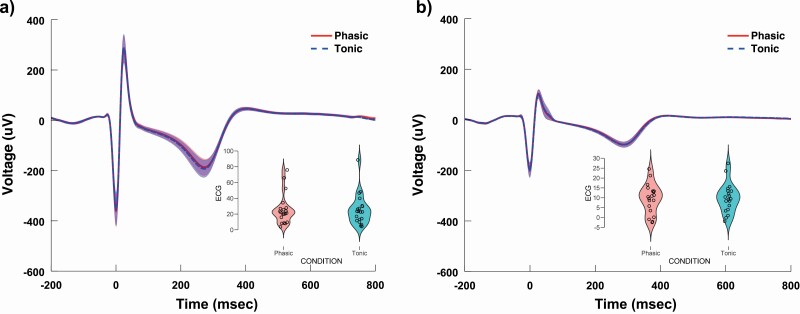
ECG amplitudes in phasic and tonic REM in Study 1 (A) and Study 2 (B). ECG waveforms were homogeneous across REM microstates, and were not associated with differences in HEPs across phasic and tonic conditions (see Sections 3.2 and 3.3). Violin plots on the right lower corner show the distribution of ECG amplitudes averaged over the time range of the positive cluster where HEP amplitudes were different across phasic and tonic REM.

First, we examined whether phasic and tonic trials differed with respect to the HEP in the time range of interest (350–650 ms). A positive cluster emerged (*t*_maxsum_ = 800.07, cluster level *p* value = 0.0442) between 571.5 and 634 ms that was most pronounced at fronto-central electrodes (Fp1, Fp2, F7, F3, F4, Fz, C3, Cz) and was not observed at temporal and posterior sites (Figure 1, A). In order to ensure that differences in HEP amplitudes were not driven by cardiac artifacts influencing scalp EEG signals, we contrasted the average ECG amplitudes of phasic and tonic conditions within the time window of the above cluster. The mean ECG amplitudes within 571.5 and 634 ms were not significantly different across phasic and tonic conditions (*t*(1,19) = 0.095, *p* = 0.93, Cohen’s *d* = 0.02). According to a repeated measures ANCOVA model, the main effect of condition (phasic vs. tonic) remained significant (*F*(1,18) = 8.03, *p* = 0.01, partial eta squared = 0.31), after controlling for phasic versus tonic differences in ECG amplitudes within the positive cluster. The contrast in mean ECG amplitudes (used as a covariate) was not significantly associated with the HEP amplitude (*F*(1,18) = 0.09, *p* = 0.35, partial eta squared = 0.05). Likewise, the contrasts of ECG amplitudes did not significantly correlate with the contrasts of HEP amplitudes (Pearson *r* = −0.28, *p* = 0.2). To compare phasic and tonic ECG beyond the significant cluster, we contrasted averaged ECG waveforms in the entire time range between –200 and 800 ms. Only 10 out of the 1,024 time points showed significant differences, exhibiting an uncorrected *p*-value below 0.05 between −200 and 194 ms relative to the R peak, and none of the time points remained significantly different after FDR correction for multiple comparisons (Figure 2, A). Finally, differences in HR and HRV (SDNN) across phasic and tonic REM conditions were not associated with differences in HEP amplitudes (HR and HEP: *r* = 0.08, *p* = 0.72; HRV and HEP: *r* = −0.03, *p* = 0.88).

### HEP differences in Study 2

In order to validate the results of the first study, we performed the same analysis on the dataset from Study 2. The cluster-based permutation tests revealed a significant difference between phasic and tonic conditions (*t*_maxsum_ = 831.02, cluster level *p* value = 0.006). A positive cluster appeared that ranged between 553.9 and 649.6 ms that extended over a larger scalp area compared to Study 1, peaked at central sites, but was observed at frontal and parietal electrode contacts as well, with the exception of centro-temporal and occipital recordings (significant cluster was observed at F7, F3, F4, Fz, C3, Cz, C4, P4, Pz electrodes). Interestingly, the shape of the HEP waveforms and the differences across conditions were remarkably similar in the two studies (Figure 1, B). Contrasting the mean ECG amplitudes of phasic and tonic conditions within the cluster’s latency range indicated no significant differences (*W*(1,18) = 79, *p* = 0.54, *d* = −0.16). Similarly to the findings of Study 1, the main effect of condition (phasic vs. tonic) remained significant (*F*(1,17) = 7.3, *p* = 0.02, partial eta squared = 0.30), after controlling for phasic versus tonic differences in ECG amplitudes within the positive cluster. The contrast in mean ECG amplitudes (used as a covariate) was not significantly related to the HEP amplitude (*F*(1,17) = 0.25, *p* = 0.61, partial eta squared = 0.01), and the contrasts of ECG amplitudes did not significantly correlate with the contrasts of HEP amplitudes (Pearson *r* = −0.12, *p* = 0.61). To compare phasic and tonic ECG beyond the significant cluster, we contrasted averaged ECG waveforms in the entire time range from –200 to 800 ms (comprising 512 time points). Amplitudes at 12 time points overlapping with the T-wave between 395.7 and 417.2 ms showed significant differences, exhibiting an uncorrected *p*-value <0.05; however, none of the time points remained significantly different after FDR correction for multiple comparisons (Figure 2, B). Similar to Study 1, differences in HR and HRV (SDNN) across phasic and tonic REM conditions were not associated with differences in HEP amplitudes (HR and HEP: *r* = 0.26, *p* = 0.28; HRV and HEP: *r* = 0.05, *p* = 0.83).

### Comparison of phasic vs. tonic HEP across Study 1 and Study 2

Since the contrast between phasic and tonic REM yielded a more robust finding in Study 2, but showed only a tendency in Study 1, we aimed to compare whether differential HEP responses between phasic and tonic REM states differed across the two studies. Therefore, we statistically compared phasic vs tonic HEP contrasts (phasic HEP – tonic HEP) between Study 1 and Study 2 in the examined time range (350–650 ms). The HEP contrasts were not significantly different according to cluster-based permutation tests (we did not identify clusters within the examined time range, see Supplementary Material, S3). In addition, we compared the contrasts of the HEP amplitudes averaged over the positive spatio-temporal clusters that emerged (and differentiated phasic and tonic REM) in Study 1 and Study 2. The phasic versus tonic HEP amplitude contrasts were not significantly different across the participants of the two studies (*t*(1,36.42) = 0.21, *p* = 0.83, Cohen’s *d* = 0.065). In addition, heart rate and heart rate variability (SDNN) did not significantly differ across the participants of Study 1 and Study 2 neither in phasic nor in tonic REM (see Supplementary Material, Table 1).

### HEP in REM microstates versus HEP in wakefulness

**Figure 3.**
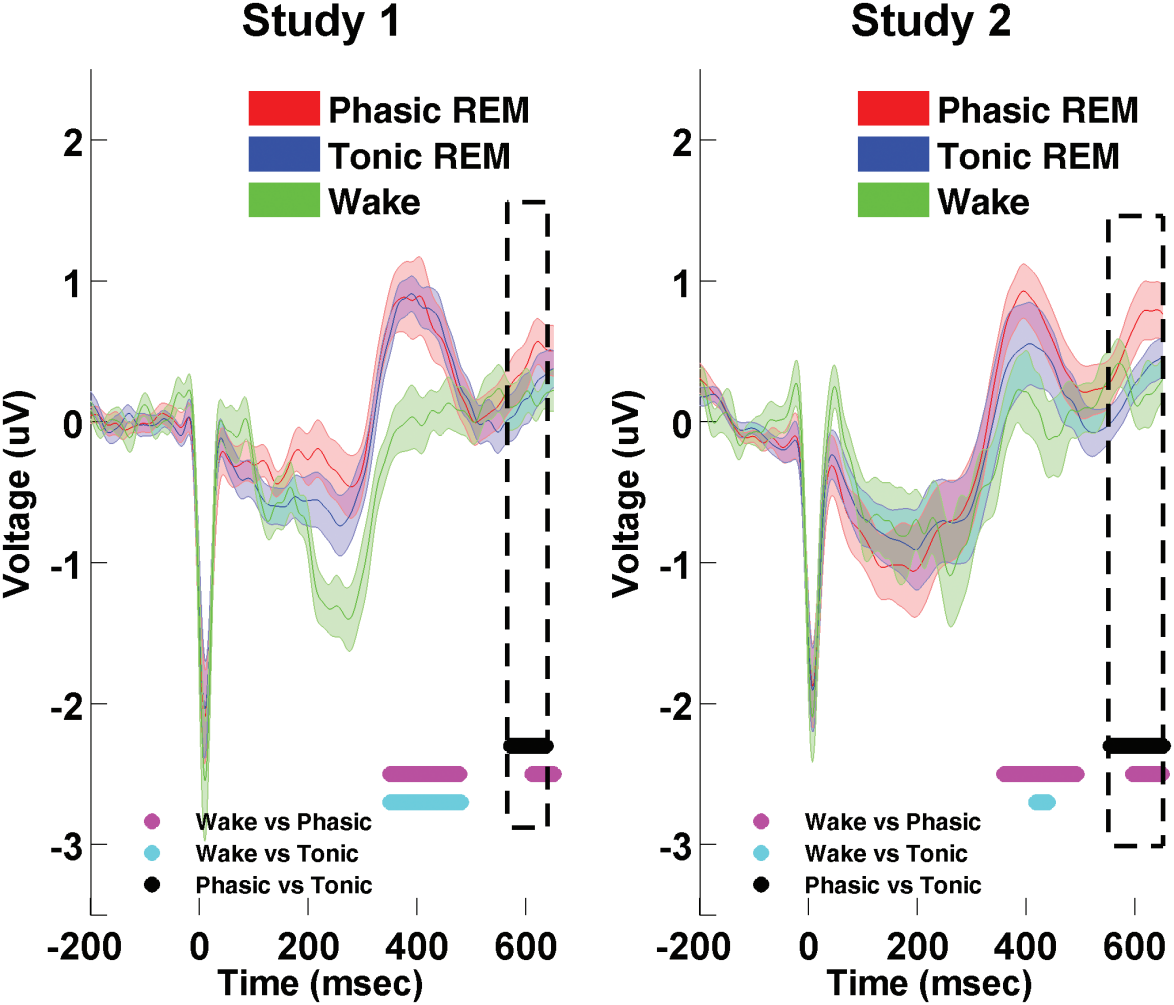
HEP (heartbeat evoked potential) waveform in REM microstates and wakefulness in Study 1 and Study 2, averaged across all electrode derivations. HEP in phasic REM sleep differed in amplitudes from wakefulness. Two clusters emerged in both studies. The first cluster was observed between 349.8 and 476.8. ms (cluster level *p* = 0.003), and between 358.6 and 491.4 ms (cluster level *p* < 0.0001), in Study 1 and Study 2, respectively. The second cluster appeared between 610.5 and 649.6 ms (cluster level *p* = 0.09) in Study 1, and between 594.9 and 647.7 ms (cluster level *p* = 0.012), in Study 2. Clusters between wakefulness and phasic REM are indicated by magenta on the time axis. HEP in tonic REM sleep showed different amplitudes compared to wakefulness, but only one cluster (indicated by light blue) appeared between 349.8 and 471.9 ms (cluster level *p* < 0.0001) and between 417.2 and 438.4 ms (cluster level *p* = 0.07) in Study 1 and Study 2, respectively. Late components of HEP did not differ across wakefulness and tonic REM sleep. The black line and the dashed rectangles indicate the cluster that emerged between the HEP waveforms of phasic and tonic REM sleep (spanning from 571.5 to 634 ms in Study 1 ms and from 553.9 to 649.6 ms in Study 2). Whereas the early clusters between REM microstates and wakefulness were confounded with ECG activity in the given time range, the late clusters appeared to be independent of ECG amplitudes within the specific time range.

Our next aim was to examine HEP in wakefulness, focusing in particular on the late potentials that differed across phasic and tonic REM. Moreover, we aimed to verify whether the HEP waveforms at this specific time range show resemblance between tonic REM and wakefulness; therefore, we contrasted the HEP waveforms across the three vigilance states. Statistical parameters of these comparisons are summarized in Table 2. Figure 3 depicts the contrast between HEPs in REM microstates and wakefulness in Study 1 and Study 2. First, we compared the HEP within the time range of interest (350–650 ms) across phasic REM sleep and wakefulness. HEP waveforms in wakefulness and phasic REM were significantly different in the examined time range. In Study 1, two clusters were apparent: extending from 349.8 to 476.8. ms, and from 610.5 to 649.6 ms. Both clusters were present at frontal, central, and parietal sites. To examine whether the appearance of the clusters could be attributed to differences in ECG activity between the awake and the phasic REM states, we contrasted the mean ECG amplitudes within the time windows of the two clusters. ECG amplitudes differed significantly in the case of the first cluster that spanned between 349.8 and 476.8 ms (*t*(1,19) = −4.32, *p* = 0.0004, *d* = −0.97), indicating that HEP amplitudes across phasic REM and wakefulness were confounded by differences in ECG amplitudes. Nonetheless, ECG amplitudes were not significantly different at the second cluster between 610.5 and 649.6 ms (*t*(1,19) = −0.11, *p* = 0.9, *d* = −0.02). The comparison of HEPs between tonic REM sleep and wakefulness yielded only one cluster between 349.8 and 471.9 ms extending throughout the scalp. Corresponding mean ECG amplitudes were also significantly different (*t*(1,19) = −3.89, *p* = 0.001, *d* = −0.87) suggesting that differences in HEP could be influenced by differences in ECG activity over this time range.

**Table 2.** HEP in REM microstates compared to wakefulness: identified clusters and cluster-level statistics across the conditions in Study 1 and Study 2.

Study 1	Phasic vs. wake		Tonic vs. wake	
	Cluster 1	Cluster 2	Cluster 1	Cluster 2
Time range (ms)	349.8–467.8	610.5–649.6	349.8–471.9	–
Cluster statistics (*t*_maxsum_ and cluster level *p*)	3031.3 (0.003)	683.54 (0.09)	4032.2 (0.002)	–
ECG difference at the cluster	Yes	No	Yes	–
Study 2	Phasic vs. wake		Tonic vs. wake	
	Cluster 1	Cluster 2	Cluster 1	Cluster 2
Time range (ms)	358.6–491.4	594.9–647.7	417.2–438.4	–
Cluster statistics (*t*_maxsum_ and cluster level *p*)	1070.9 (0.0001)	476.11 (0.012)	135.79 (0.07)	–
ECG difference at the cluster	Yes	No	Yes	–

In Study 2, the comparison of phasic REM and wakefulness yielded two clusters, the first spanned between 358.6 and 491.4 ms in centroparietal derivations, and the second between 594.9 and 647.7 ms involving all the electrodes except the lateral chain. The first cluster appeared to be related to differences in ECG amplitudes to some extent, since the average ECG amplitudes of the first cluster between phasic REM and wakefulness differed on a trend level (*t*(1,15) = −1.5, *p* = 0.078, *d* = −0.37), whereas average ECG amplitudes of the second cluster was not significantly different across the two conditions (*t*(1,15) = −0.24, *p* = 0.4, *d* = −0.06). The contrast of HEPs between tonic REM and wakefulness produced a narrow cluster between 417.2 and 438.4 ms that showed a trend. Average ECG amplitudes between tonic REM and wakefulness showed a trend (*t*(1,15) = −1.23, *p* = 0.12, *d* = −0.30). (See Table 2 for more details.)

In sum, in both studies, HEP amplitudes in phasic REM differed, but HEPs in tonic REM were not distinguishable from HEPs in wakefulness in the time range (between 600 and 650 ms) that also differentiated phasic and tonic REM sleep (see Figure 3 and Supplementary Figure S1 showing the averaged ECG waves).

### Are the differences between vigilance states related to heartbeats?

In order to verify whether the identified differences across vigilance states are specifically locked to heartbeats or merely reflect differences in oscillatory activity between REM microstates and resting wakefulness, we performed the same analyses on surrogate heartbeats. In Study 1, 99 % of the analyses (all but one) on the surrogate data yielded lower sum of *t*-values than the original cluster statistic contrasting phasic and tonic HEPs. In Study 2, we did not find any cluster obtained from the analyses of the surrogate datasets that had a larger or equal sum of *t*-values than the cluster statistic comparing the original data. These findings indicate that the observed differences in EEG amplitudes were particularly linked to the heartbeats and were not confounded by general differences in cortical oscillations (see Supplementary Material S4 for an illustration).

## Discussion

Our findings indicate that neural responses to cardiac signals are not uniform across REM microstates: differences in HEP amplitude between tonic and phasic REM were observed in both studies. Importantly, the two separate analyses yielded strikingly similar HEP waveform patterns for each REM microstate, while the differences between the two states were identified in the same latency range (compare Figure 1, A and B). Although the effect appeared to be more robust in Study 2, differential (phasic vs tonic) HEP waveforms were not statistically different across the two studies in the examined time range (see Supplementary material, S3). These differences in the late components of HEPs were apparently not related to ECG artifacts (see Figure 2, A and B that show an absence of significant differences between the average ECG signals). Furthermore, based on additional analyses comparing HEP waveforms in REM microstates with HEPs in resting wakefulness, the late HEP component differentiating the two microstates was nominally (Study 1) and significantly (Study 2) different across wakefulness and phasic REM sleep but not across wakefulness and tonic REM sleep (Figure 3). Accordingly, the HEP waveform in tonic REM within these late potentials (~550–650 ms) resembled the HEP waveform of resting wakefulness in both studies.

Although the results of the two studies reported here leave little room for doubt concerning the differential HEP modulation between tonic and phasic REM, the interpretation of these HEP differences is not straightforward. Our best evidence for the existence of the HEP comes from intracranial recordings that showed clear, artifact-free, phasic neural responses time-locked to the ECG R-peak [55, 65–67]; for a recent review see [28]). These recordings detected heartbeat evoked activity in the somatosensory cortex [66], and in the right anterior insula [55, 65, 67]. Most HEP studies, however, measure cortical activation indirectly using scalp EEG or MEG. A careful review of the relevant literature reveals a striking variability in HEP waveform pattern, topography, and temporal localization. This disparity across HEP studies may reflect not only the multifaceted nature of HEP’s physiological origins, but also the degrees of freedom in methodological choices, such as the artifact correction method, the number of electrodes, the location of the reference electrode and the a priori selection of specific ROIs. As a result, there is still no consensual interpretation of modulations in HEP amplitude, as in the case, for example, of the P300 ERP component, where a positive deflection from the baseline measured in parietal regions is unequivocally interpreted as a “larger” P300 [68]. In the HEP literature, “larger” HEP responses have been identified both with positive [31, 58, 69] and negative amplitudes [32, 33, 70, 71].

Given the current lack of congruency, here we do not attempt to offer an interpretation of the observed differences in terms of “larger” or “increased” HEP based on the amplitude of the responses. Nevertheless, the contrast between REM and Wake HEP allows us to extend the interpretation of our findings based on the resemblance between tonic and wake conditions, and their difference with the phasic condition in the specific time range that differentiated phasic from tonic REM sleep (Figure 3). Thus, our results are in agreement with previous studies where tonic REM appeared as an intermediate state between phasic REM and wakefulness with respect to external information processing. Specifically, existing evidence suggests that while environmental alertness is largely reduced during phasic REM sleep, external processing is partially reinstated during tonic REM periods. Wehrle et al. [15] observed preserved BOLD activity in the auditory cortex in response to acoustic stimulation during tonic REM periods that to some extent resembled the patterns found in wakefulness. In sharp contrast, no similar activity in the corresponding cortical regions were observed during phasic REM periods. In line with these findings, event-related potentials resembling the awake P300 potential could be elicited specifically during tonic, but not in phasic REM using paradigms that contrasted evoked responses to frequent (standard) tones with the responses to rare (deviant) tones [19–21]. Furthermore, a more recent study observed the selective processing of informative speech (compared to meaningless speech) during tonic REM, whereas such informational selectivity disappeared in phasic REM periods [18]. In line with these findings, our results suggest that cardiac cycle-related neural activity in tonic REM is closer to that of resting wakefulness, than neural activity in phasic REM. Accordingly, the present findings provide further support for the assumption of tonic REM sleep as a relatively more “open state” to environmental inputs, and extends this notion to the domain of interoceptive signal processing.

We should note that differences in HEP across REM microstates were observed in late potentials in both studies. Although the HEP was usually reported to occur earlier [31, 56], the modulation of its amplitude was also observed at later time points (after 500 ms) in several studies [58, 72, 73]. On the other hand, evoked potentials (e.g. the P300-like response) in REM sleep were reported to occur with a longer peak latency in comparison with similar evoked potentials in wakefulness [19, 74]. In addition, HEPs in resting wakefulness differed from the HEP waveforms of both REM states in an earlier time window (peaking around 400 ms). Nevertheless, the analyses of ECG amplitudes also showed differences across wakefulness and REM microstates in that time range that overlapped with the T-wave (see upplementary material, S2). In wakefulness the T-wave expressed a larger peak, steeper ascending and descending limbs, and shorter interval, resulting in amplitude differences that were confounded with the HEP differences (across wake and REM microstates) identified over these earlier time ranges. Therefore, we retain from interpreting these differences further since HEP differences over these time points appear to be influenced by the proximity of the T-wave contaminating the EEG signal. Further studies should verify whether HEP waveforms differ across wakefulness and REM microstates regardless of differences in ECG amplitudes.

Our results are also in accord with the findings that indicate changes in HEP with state or trait-dependent variations of arousal during wakefulness [38, 39]. Moreover, our findings are in line with the gradual attenuation of the HEP as we move from wakefulness to Stage 1 sleep, REM sleep, and finally deeper sleep stages (Stage 2 and SWS), indicating that HEP amplitudes are sensitive to changes in alertness and arousability during sleep [35]. Regarding spontaneous oscillatory activity, tonic periods exhibit a relative increase within the high alpha (10–14 Hz) and beta (15–28 Hz) frequency bands compared to phasic periods as observed in scalp EEG [41, 61, 75, 76] and intracerebral recordings [13]. Although the neural correlates of increased high alpha and beta frequency power during REM sleep remain to be explored, such oscillations are also predominant and index alertness in wakefulness [77, 78], during which they were associated with the activity of a cingulo-opercular network maintaining arousal and vigilance [79, 80]. Therefore, spontaneous oscillatory activity in REM microstates also indicate an increase in arousal-related processes (and hence in environmental alertness) during tonic as compared to phasic REM periods.

The processing of internal signals, including thermoception, nociception, and visceroception is an integral aspect of the sleeping brain [24]. Similarly to acoustic processing (the most studied domain in the field), sensory thresholds for interoceptive inputs (e.g. nociceptive stimuli) seem to be increased as a function of sleep depth [25]. Nevertheless, cortical responses to internal signals persist during sleep, and might be facilitated during transient states of reinstated environmental alertness and increased arousal. The putative link between increased arousal and the HEP was supported in studies that showed increased HEP after the experimental induction of arousal [38], and increased HEP (during resting wakefulness) in insomnia patients compared to good sleepers [39]. Since hyperarousal in insomnia is not limited to the wakeful period before falling asleep but seems to characterize sleep, and more specifically REM sleep [81, 82], future studies might examine alterations of HEP in insomnia disorder and other NREM and REM parasomnias during different sleep stages, and explore its utility as a predictor of sleep fragmentation.

The so-called restorative functions of sleep are accomplished under longer periods of environmental disconnection and largely diminished behavioral responsiveness, often termed as off-line states [3, 83, 84]. At the same time, however, the sleeping brain is required to maintain sensory processing to some extent as a complete detachment of the surroundings would have evidently reduced chances of survival through natural selection [2, 3, 15]. A growing number of studies indicate that during non-REM (NREM) sleep, the most dominant state of human sleep, these antagonistic needs are coordinated by the periodic fluctuation between stabile sleep states and periods of increased susceptibility to external inputs [1, 3–5]. The alternation of phasic and tonic periods may share a similar role in REM sleep [22]. Here, we showed that differences in sensory processing across phasic and tonic microstates might not only be limited to exteroception [15, 18, 19, 21], but also influence the processing of internal, bodily signals. Hence, our results are the first to indicate that the partially reinstated environmental alertness during tonic REM is also observed with regards to the processing of cardiac signals. We may speculate that increased cortical processing of cardiac activity may reinstate bodily representations that could also facilitate external processing [28, 85] and prepare the organism for motor control following awakening. On the other hand, the brain appears to be less sensitive to cardiac afferent signals during phasic REM periods, when neural processing is temporarily liberated from the constraints of external stimuli, which presumably contributes to the peculiar sensorimotor experiences of dreaming.

## Supplementary Material

zsab100_suppl_Supplementary_Materials

## Data Availability

Anonymized data and analysis scripts can be found on the project’s OSF page: https://osf.io/2vptx/?view_only=16c210651ae846dc95a8f50ef4ac3d68
