# Supplementary material for: Cortical monitoring of cardiac activity during rapid eye movement sleep: the heartbeat evoked potential in phasic and tonic rapid-eye-movement microstates
Source: Sleep. 2021 Apr 17;44(9):zsab100. doi: 10.1093/sleep/zsab100 (PMC8633618; doi:10.1093/sleep/zsab100)
Supplement: zsab100_suppl_Supplementary_Materials [file zsab100_suppl_supplementary_materials.docx]

**Cortical monitoring of cardiac activity during rapid eye movement sleep: the heartbeat evoked potential in phasic and tonic REM microstates**

*Péter Simor^1,2,3*^, Tamás Bogdány^1*^, Róbert Bódizs^2,4^, Pandelis Perakakis^5^*

*1 Institute of Psychology, ELTE, Eötvös Loránd University, Budapest, Hungary.*

*2 Institute of Behavioural Sciences, Semmelweis University, Budapest, Hungary.*

*3 UR2NF, Neuropsychology and Functional Neuroimaging Research Unit at CRCN - Center for Research in Cognition and Neurosciences and UNI - ULB Neurosciences Institute, Université Libre de Bruxelles (ULB), Brussels, Belgium.*

*4 National Institute of Clinical Neurosciences, Budapest, Hungary*

*5 Department of Social, Organisational, and Differential Psychology, Complutense University of Madrid, Spain*

*6 Brain, Mind, & Behavior Research Center, University of Granada, Spain*

Corresponding author:

*Péter Simor, Ph.D.*

*Institute of Psychology, ELTE Eötvös Loránd University, Budapest, Hungary.*

*1064 Budapest, Izabella utca 46.*

[*simor.peter@ppk.elte.hu*](mailto:simor.peter@ppk.elte.hu)

*+36302755968*

***the authors contributed equally to this work.**

**Supplementary Materials**


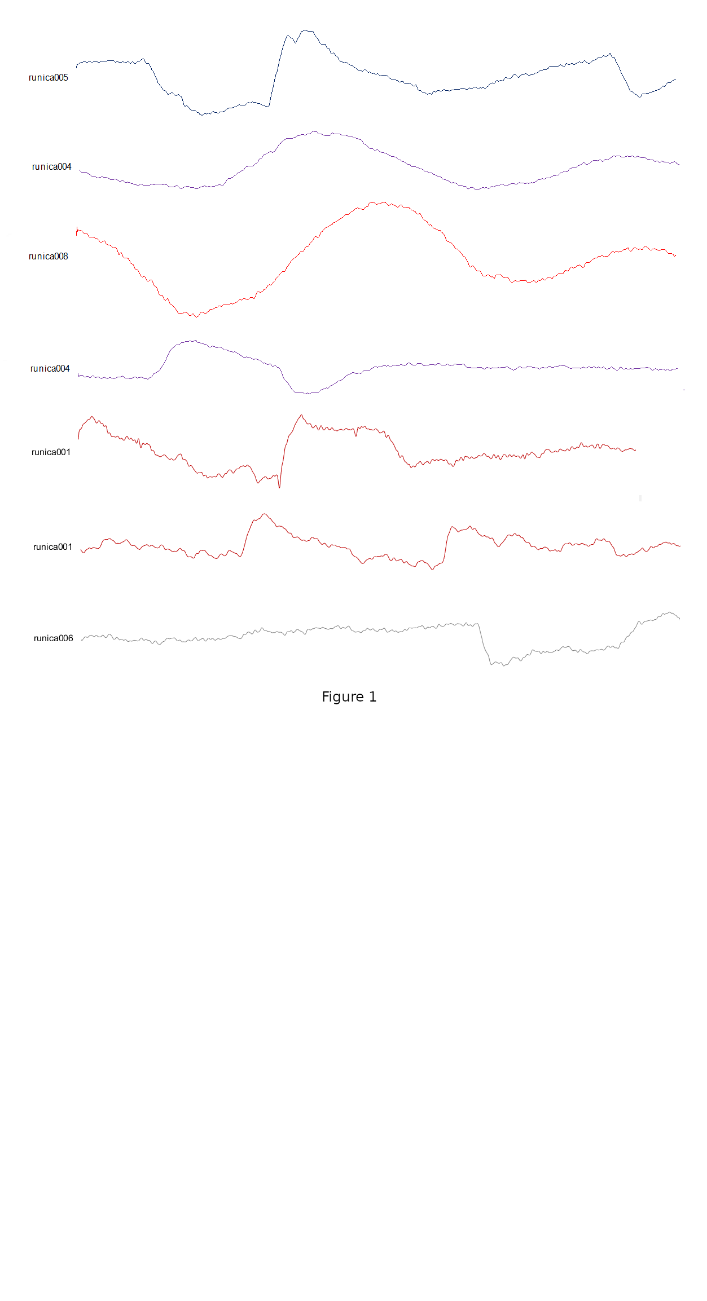

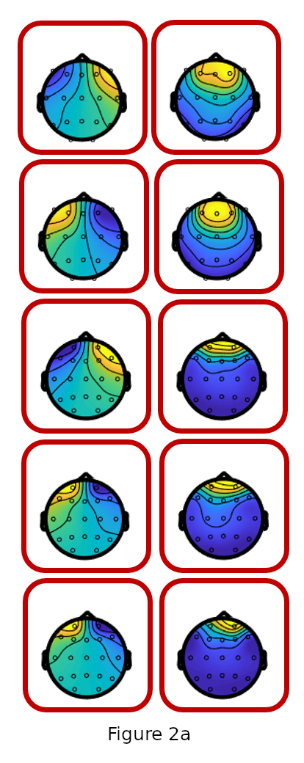

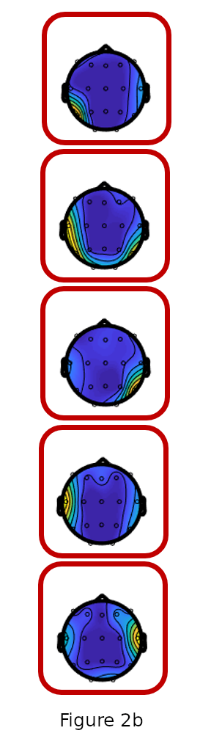


**S1.** Independent component analysis (ICA) of the concatenated phasic REM, tonic REM and resting wake segments was performed to identify eye movement artifacts using EEGLAB and Fieldtrip routines (Delorme and Makeig, 2004; Oostenveld et al., 2011). Method was set to 'runica', without performing baseline correction (demean='no'). The number of the removed components were 2.1 (SE=0.31) in the first and 2.25 (SE=1.02) in the second study (total number of components were 19 in both studies, mostly two, maximum four components were removed). Independent components representing rapid eye movements were detected semi-automatically and were identified by inspecting the waveforms during the three vigilance states, as well as their topographical distribution (Campos Viola et al., 2009). Typical pattern for eye movements shown on Figure 1. were targeted for component removal. Figure 2a and 2b shows typical topographies selected for removal (eye and movement artefact respectively).


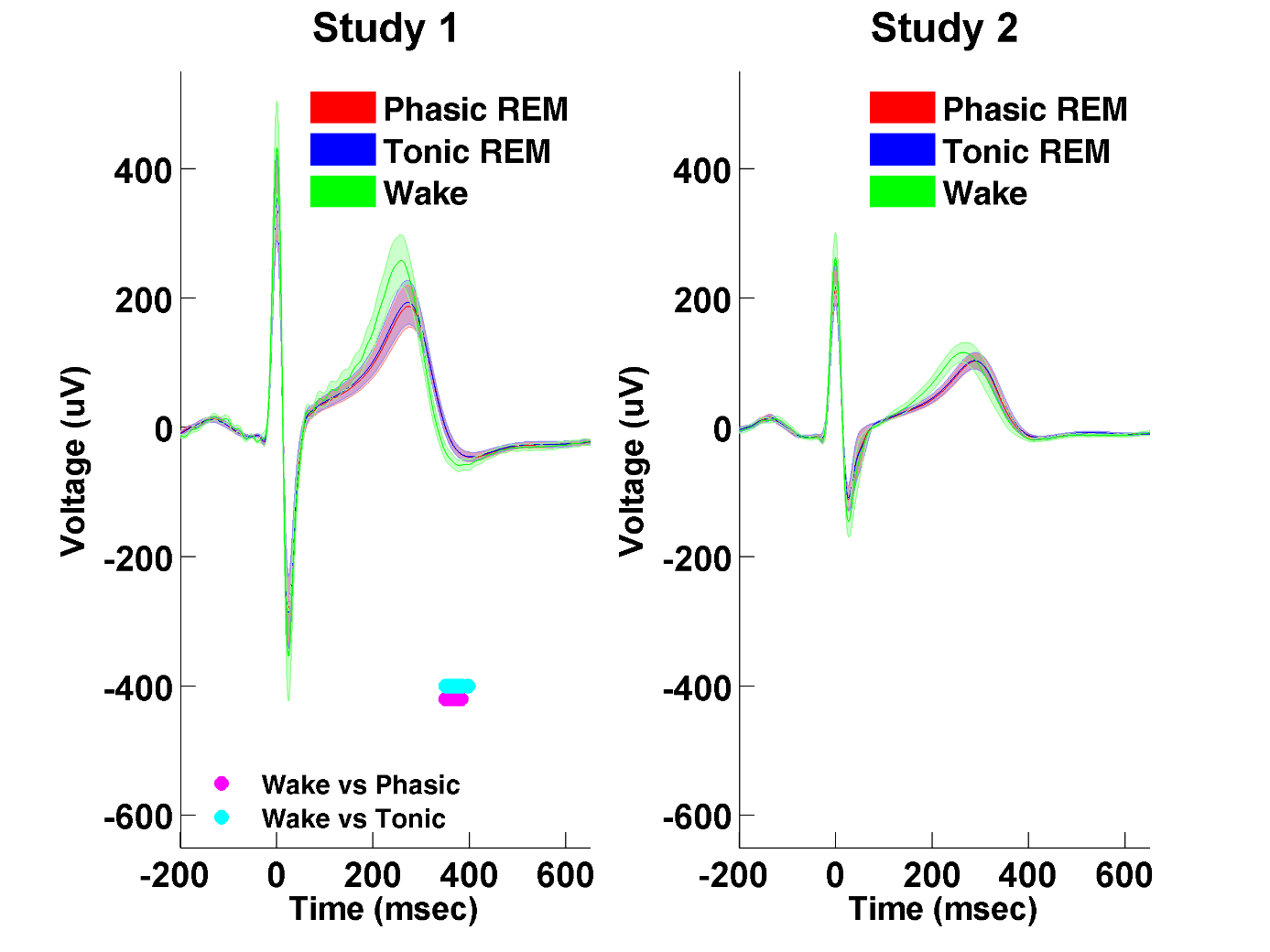


**S2.** Averaged ECG amplitudes in REM microstates and resting wakefulness in Study 1 (N = 20) and Study 2 (N = 16). Time points showing significant differences across conditions after FDR correction are marked by light blue (Wakefulness vs Phasic REM), and magenta (Wakefulness vs Tonic REM) colors.

**
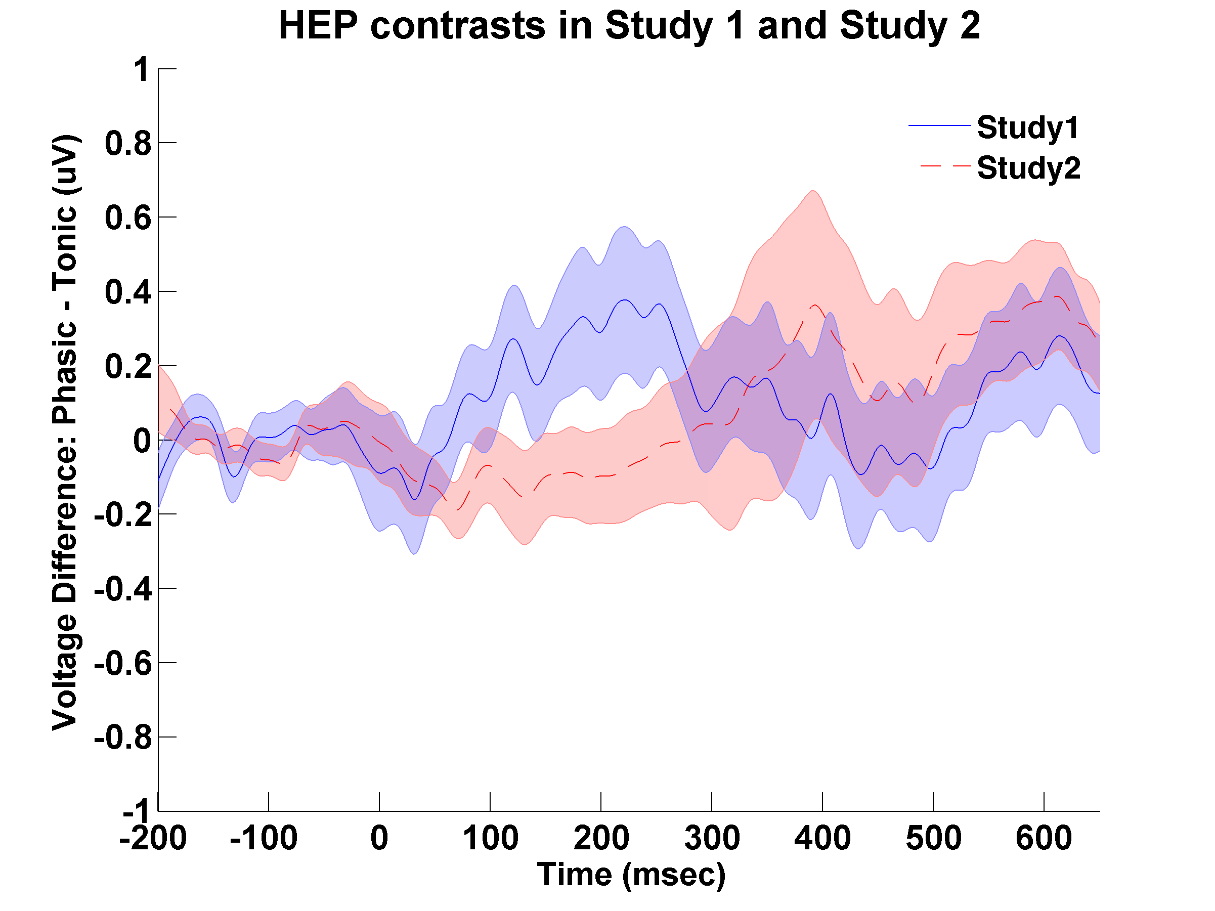
**

**S3.** Phasic and Tonic HEP contrasts in Study 1 (N = 20) and Study 2 (N = 19). The lineplots indicate the difference between Phasic and Tonic HEP averaged across electrodes F7, F8, F3, F4, Fz, C3, C4, Cz, T3, T4, T5, T6, P3, P4, Pz, O1, and O2. (Fp1 and Fp2 were not included in this comparison, since these electrodes were only present in Study 1). No significant differences emerged between the two studies within the examined time range (350 and 650 ms).

PHASIC REM


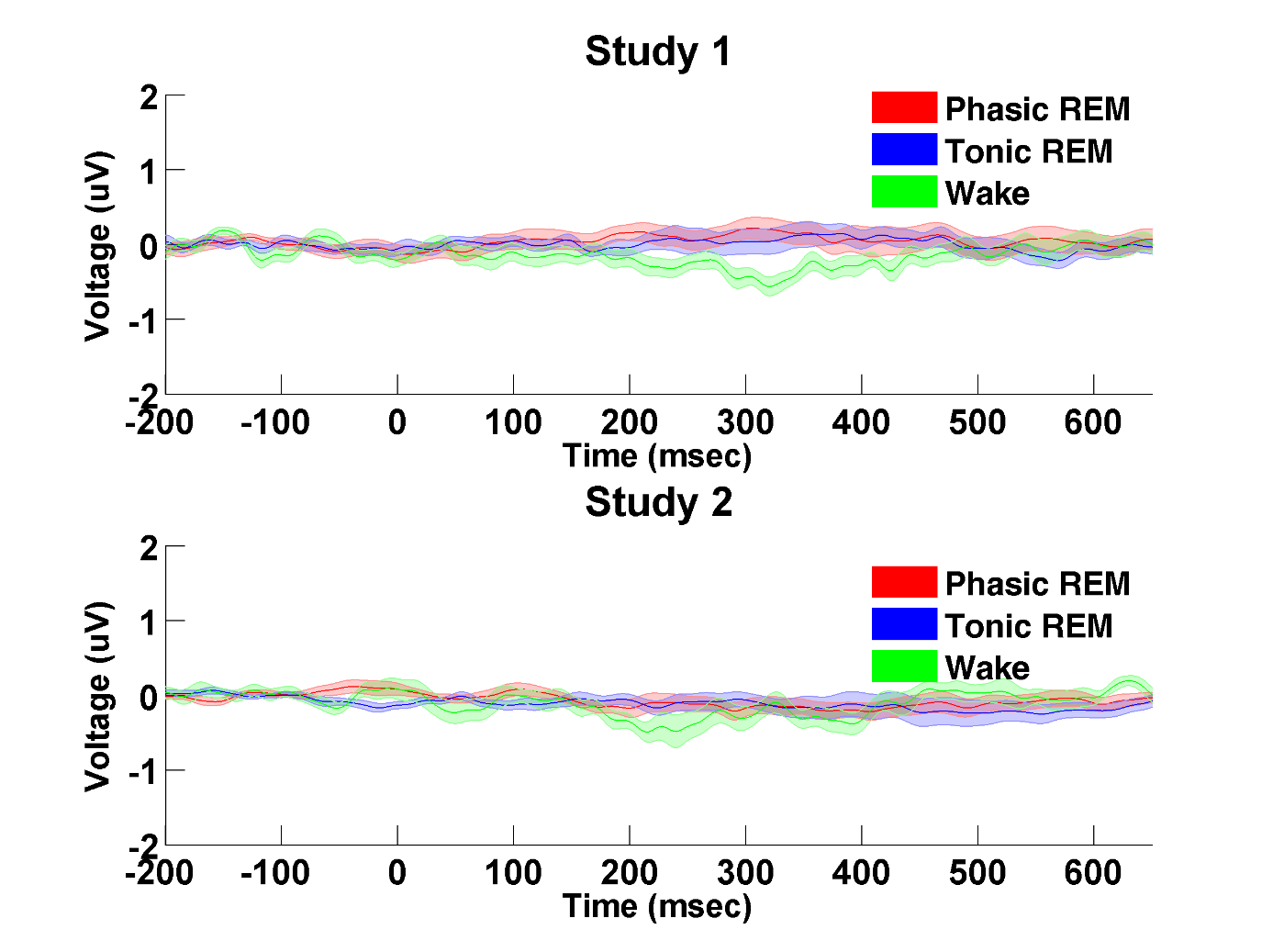


**S4.** An example of averaged EEG trials time locked to surrogate heartbeats in Study 1 and Study 2. Surrogate R-peaks were defined as random time points similar in number and inter-beat intervals to the observed R-peaks. 1000 ms trials (segments between -200 ms and 800 ms relative to the surrogate R-peaks) were averaged for each participant in phasic and tonic REM sleep, and compared with cluster-based permutation testing. The same procedure was repeated 100 times, and the distribution of the sums of t-values were extracted. In Study 1, 99 % of the analyses on the surrogate data yielded lower sum of t- values than the original cluster statistic contrasting phasic and tonic HEPs. In study 2, we did not find any cluster obtained from the analyses of the surrogate datasets that had a larger or equal sum of t-values than the cluster statistic comparing the original data. These findings indicate that the observed amplitude (HEP) differences across phasic and tonic REM were particularly linked to the occurrence of heartbeats.

1. Phasic REM

B) Tonic REM

**Table S1.** Statistical group comparisons of heart rate and variability across participants of Study 1 (N = 20) versus Study 2 (N = 19). P-values were adjusted by FDR to correct for multiple comparisons.

| **Independent Samples T-Tests** | | | | | | | | | |
| --- | --- | --- | --- | --- | --- | --- | --- | --- | --- |
|  | | **t** | | **df** | | **Adjusted p** | | **Cohen's d** | |
| HR phasic |  | 0.7944 |  | 37.0000 |  | 0.561 |  | 0.2545 |  |
| SDNN phasic |  | 0.5866 |  | 37.0000 |  | 0.561 |  | 0.1879 |  |
| HR tonic |  | 0.6404 |  | 37.0000 |  | 0.561 |  | 0.2052 |  |
| SDNN tonic |  | 1.8540 |  | 37.0000 |  | 0.286 |  | 0.5939 |  |
|  | | | | | | | | | |
| *Note.*  Student's t-test. | | | | | | | | | |

**References**

Delorme A, Makeig S. EEGLAB: an open source toolbox for analysis of single-trial EEG dynamics including independent component analysis. *Journal of neuroscience methods*. 2004;134(1):9–21.

Oostenveld R, Fries P, Maris E, Schoffelen J-M. FieldTrip: open source software for advanced analysis of MEG, EEG, and invasive electrophysiological data. *Computational intelligence and neuroscience*. 2011;2011:1.

Campos Viola F, Thorne J, Edmonds B, Schneider T, Eichele T, Debener S. Semi-automatic identification of independent components representing EEG artifact. *Clinical Neurophysiology*. 2009;120(5):868-877. doi:10.1016/j.clinph.2009.01.015
